# Studies on the Dual Activity of EGFR and HER-2 Inhibitors Using Structure-Based Drug Design Techniques

**DOI:** 10.3390/ijms19123728

**Published:** 2018-11-23

**Authors:** Rafaela Molina de Angelo, Michell de Oliveira Almeida, Heberth de Paula, Kathia Maria Honorio

**Affiliations:** 1 School of Arts, Sciences and Humanities (EACH), University of São Paulo (USP), São Paulo 03828-000, Brazil; rafaela.molina.angelo@usp.br (R.M.d.A.); heberth.paula@ufes.br (H.d.P.); 2 Center for Natural and Human Sciences (CCNH), Federal University of ABC (UFABC), Santo André 09210-580, Brazil; michelloliveira02@gmail.com; 3 Department of Pharmacy and Nutrition, Center of Exact, Natural and Health Sciences, Campus of Alegre, Federal University of Espirito Santo—CCENS, Alegre 29500-000, Brazil

**Keywords:** cancer, HER-2, EGFR, drug design, docking, CoMFA, CoMSIA, dual inhibitor

## Abstract

HER-2 and EGFR are biological targets related to the development of cancer and the discovery and/or development of a dual inhibitor could be a good strategy to design an effective drug candidate. In this study, analyses of the chemical properties of a group of substances having affinity for both HER-2 and EGFR were carried out with the aim of understanding the main factors involved in the interaction between these inhibitors and the biological targets. Comparative analysis of molecular interaction fields (CoMFA) and comparative molecular similarity index analysis (CoMSIA) techniques were applied on 63 compounds. From CoMFA analyses, we found for both HER-2 (r^2^ calibration = 0.98 and q^2^_cv_ = 0.83) and EGFR (r^2^ calibration = 0.98 and q^2^_cv_ = 0.73) good predictive models. Good models for CoMSIA technique have also been found for HER-2 (r^2^ calibration = 0.92 and q^2^_cv_ = 0.74) and EGFR (r^2^ calibration = 0.97 and q^2^_cv_ = 0.72). The constructed models could indicate some important characteristics for the inhibition of the biological targets. New compounds were proposed as candidates to inhibit both proteins. Therefore, this study may guide future projects for the development of new drug candidates for the treatment of breast cancer.

## 1. Introduction

There are two important biological targets related to breast cancer: Human Epidermal Growth Factor Receptor 2 (HER-2) and Epidermal Growth Factor Receptor (EGFR) [1]. Overexpression of ErbB (this abbreviation is derived from the name of a viral oncogene to which these receptors are homologous: erythroblastic leukemia viral oncogene) family members has implicated in many human cancers, and HER-2 expression is predictive of recurrence of human disease and prognosis. Inhibitors of the kinase domain of EGFR and HER-2 have been approved for the treatment of cancer, for example, erlotinib, lapatinib and trastuzumab [2]. Receptors of the HER (ErbB) family are critical for the development of various organs and systems. When activated, these receptors bind to dimers, transphosphorylate and become capable of transducing intracellular signals that can affect cell growth, the inhibition of apoptosis, the migration and invasiveness, and angiogenesis, among other processes that lead to progression of malignant tumors [3]. The simple overexpression of HER-1 (EGFR) does not transform cells, since the HER1:HER1 dimer is only capable of being transphosphorylated when one of its extracellular ligands is coupled in its active site. HER-2, for which an extracellular ligand has not yet been described, may spontaneously form dimers, a characteristic conferred by the peculiar structure of its extracellular portion.

Usually, two copies of the HER-2 gene are found in each cell, which must produce an adequate amount of protein on the cell surface. In breast cancer, one can find 25–50 copies of the HER-2 gene and an increase of the protein amount by 40–100 times, resulting in 2 million receptors expressed in the tumor cell; the amplification is what defines a subtype of cancer, with a gene signature, and is maintained during the cancer progression [4].

The protein, after binding to a ligand, is activated by means of homo- or heterodimerization, leading to a cascade of events that activate its tyrosine kinase domain and promoting the rapid cell growth, differentiation, survival and migration associated with HER-2 positive breast cancer [5]. Thus, the HER2:HER2 dimer can be transphosphorylated independently of the absence of ligand, stimulating morphological transformation and cell growth, either in its mutated form or not [6]. There is evidence of a preferred binding partner between HER-2 and EGFR, and the HER-2/EGFR heterodimer shows an increase in the relative signaling potency for the EGFR homodimers. In contrast to most tyrosine kinase receptors, the loop phosphorylation is not required for the kinase activation, whereas kinase is intrinsically self-inhibitory in the cell [3].

It has been suggested that HER-2 can play an important role in the oncogenic activity of EGFR. Preclinical studies have shown that both EFGR and HER-2 act in a synergetic way in the cellular transformation [7]. HER-2 is the main and most common partner on heterodimerization of EGFR [8]. Thus, HER-2 contributes to an extension of the EGFR activity by improving the affinity by ligands [8], reducing its degradation [9] and increasing its predisposition recycling [10]. Moulder et al. showed that specific EGFR inhibitors can reduce the HER-2 signalization and the growth of breast cancer cells that overexpress HER-2 [11]. Lapatinib is a good dual inhibitor of EGFR and HER-2 and it is approved by FDA in combination with capecitabine for the treatment of patients in advanced stage or metastatic breast cancer who have not responded to other drugs. However, not all cells that overexpress HER-2 also respond to the treatment with lapatinib and some patients have presented resistance to this drug [12]. Thus, the proposal of new inhibitors of both EGFR and HER-2 can be more effective than simply targeting one of them alone.

Several studies attempt to inhibit the biological targets under study and one way to study the interaction processes between HER-2/EGFR and inhibitor molecules is employing molecular modeling methods, which are often employed in medicinal chemistry [6,13,14,15,16]. Using these techniques, it is possible to identify the interactions that occur between bioactive molecules and biological receptors. To quantify the structure and activity relationships of diverse compounds, two important techniques have been widely employed elsewhere: Comparative Molecular Fields Analysis (CoMFA) and Comparative Molecular Similarity Index Analysis (CoMSIA) [17,18,19,20,21]. The main objective of this study was to assess the interactions that occur between HER-2/EGFR and dual inhibitors (acting on both HER-2 and EGFR) and, consequently, understand their inhibition mechanisms and propose new models of drugs to treat related diseases, such as breast cancer.

## 2. Results

### 2.1. Redocking and Docking Analyses

**Figure 1:**
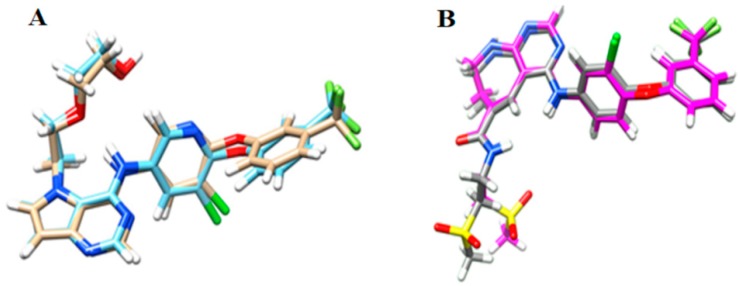
Redocking results for: (**A**) HER-2; and (**B**) EGFR. RMSD value for HER-2 = 0.464 Å and RMSD value for EGFR = 1.213 Å.

The best parameters chosen from redocking of HER-2, which presented the lowest RMSD (Root Mean Square Deviation) values, were: (i) definition of the active site within 5 Å of the crystallographic ligand; and (ii) Goldscore as the scoring function used to classify (rank) the conformations generated. For EGFR, the parameters chosen were: (i) definition of the active site within 5 Å of the crystallographic ligand; and (ii) Chemscore as the scoring function employed to classify (rank) the generated conformations. Figure 1 shows the redocking results and the RMSD values for HER-2 and EGFR, respectively. Using these parameters, molecular docking simulations for all compounds in the dataset were carried out and the best conformation of each inhibitor at properly biological target (HER-2 and EGFR) was chosen according to the greatest number of interactions with the main residues in the active site of HER-2 and EGFR.

**Figure 2:**
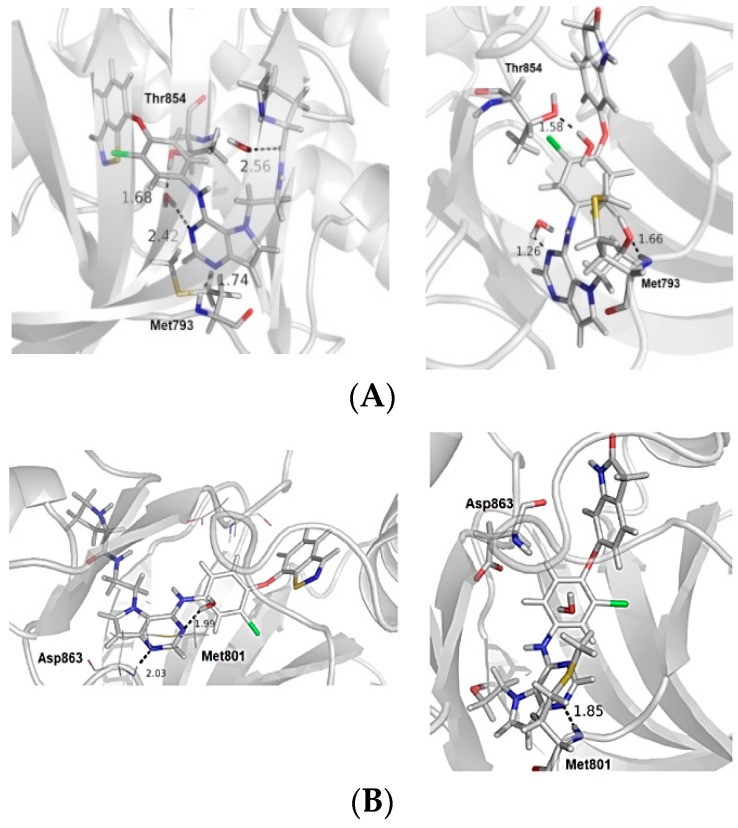
(**A**) The most and the least active compounds at the EGFR active site, along with the main residues (Met793 and Thr854) and two structural water molecules. (**B**) The most and least active compounds at the HER-2 active site, along with the main residues (Met801 and Asp863) and a structural water molecule. The numbers refer to distance between some residues and the ligands. We modified the figures now.

**Figure 3:**
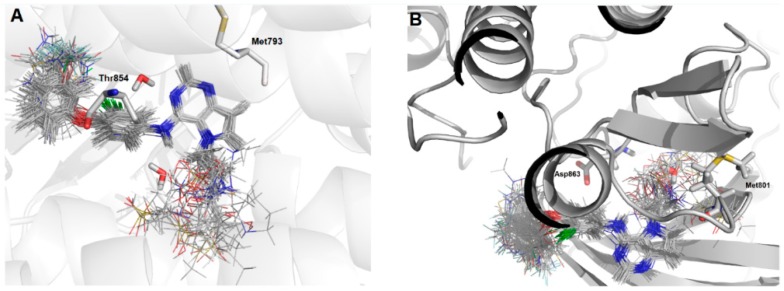
Molecular alignment of the compound set at the active site of: (**A**) EGFR; and (**B**) HER-2. Images generated using Pymol software (The PyMOL Molecular Graphics System, Version 2.0 Schrödinger, LLC.).

Figure 2 shows the main interactions between the most and least active compounds in each biological target (EGFR and HER-2). Figure 3 displays the molecular alignment obtained for all compounds at each biological target. Considering that the CoMFA and CoMSIA techniques are strictly dependent of the molecular alignment, in Figure 2 and Figure 3 we can consider the docking methodology was carried out successfully, since the main interactions described in the literature were observed in the active site of each target.

### 2.2. Construction and Validation of the CoMFA and CoMSIA Models

#### 2.2.1. CoMFA

Initially, a CoMFA model with the standard parameters was constructed. After this step, we used the option “region focus” with the aim of refining the statistical results. Table 1 displays the main results for the best models (according to q^2^_LOO_ values). In CoMFA model construction, two things caught the attention. First, the option “region focus” improved the statistical quality of the models. Finally, the proportion of steric/electrostatic contribution is inversed for both receptors, i.e., there is a greater electrostatic contribution for the HER-2 model and a greater steric contribution for the EGFR model.

**Figure 4:**
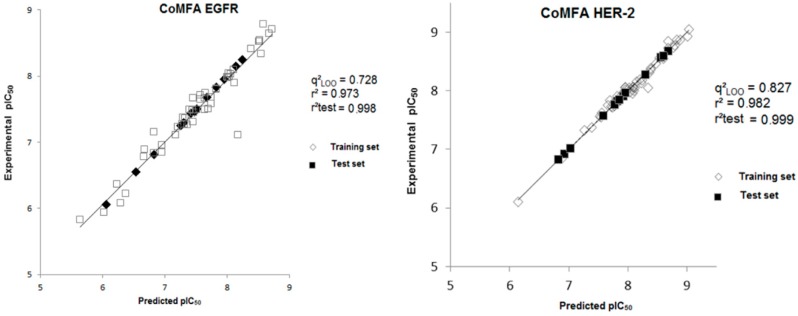
Experimental versus predicted pIC_50_ values for the training and test sets obtained from the CoMFA model for both biological targets.

Figure 4 shows the plot that correlates experimental and predicted biological data. The predicted values are in agreement with the experimental data, indicating the statistical robustness of the 3D models. Next, the external validation step was performed, in which the activity of the test compounds was predicted from the CoMFA constructed models. Table 2 presents the predicted activity values for the test set compounds, as well as their residue values.

In Table 2, we can observe that all calculated residues (experimental pIC_50_-predicted pIC_50_) for the models are lower than 0.03 log units and the highest prediction error obtained from the CoMFA model using the test set is 0.83/0.08 for HER-2 and 0.73/0.13 for EGFR. The mean value of the calculated residues for the CoMFA model is 0.005 for HER-2 and 0.008 for EGFR.

**Figure 5:**
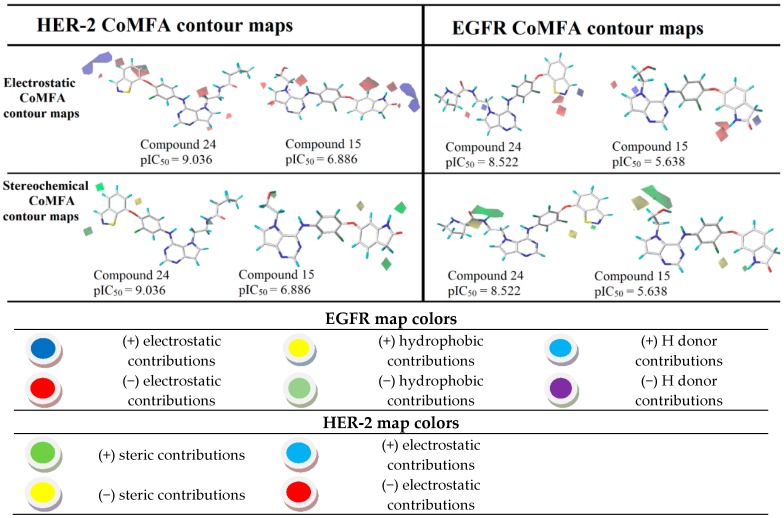
CoMFA contour maps for the most and the least active compounds (EGFR and HER-2).

Another technique used for the validation of the models is progressive scrambling (Figure S1, Supplementary Materials), which is implemented in Sybyl 8.1. This test was performed based on the biological activity values for HER2 and EGFR (bins) and, for each bin variation, 100 new scrambling models were generated with the aim of determining the sensitivity of the model to chance correlations. Therefore, the sensitivity index (slope value between q^2^ vs. r^2^) for HER2 is equal to 1.17 and 0.93 for EGFR (the values accepted for this index range from 0.8 to 1.2). From these statistical analyses, we can assert that these models were not obtained by chance correlations, i.e., these models can be considered statistically reliable. After all statistical analyses of the 3D constructed models taking into account the robustness and the predictive ability, 3D contour maps were generated for the most active ligand (24) and the least active one (15), as shown in Figure 5.

#### 2.2.2. CoMSIA

The strategy used for the CoMSIA analyses was similar to that used in the CoMFA ones. The first step of this analysis was the construction of several models using the standard configurations. This technique has several descriptors (electrostatics (E), stereochemical (S), hydrophobic (H), hydrogen-bonding acceptors (A) and hydrogen bonding donor (H)). These fields were combined in pairs, trios, quartets and a combination containing all fields.

The best model obtained for both targets presented satisfactory results, and the best value of the internal validation coefficient was 0.502 for the combination of E/S fields for HER-2 and E/H/D (0.457) for EGFR. Even this analysis presenting acceptable values of q^2^_LOO_, the “region focus” technique was used with the intention of optimizing the best model obtained previously. Table 3 presents the statistical parameters obtained with the “region focus” option. As expected, the combination that presented a greater value of the internal validation coefficient was the combination between the electrostatic and electrostatic (S/E) similarity fields for HER-2 and electrostatic, hydrophobicity and hydrogen bonding donors (E/H/D) for EGFR. The values obtained for this model of HER-2 was q^2^_LOO_ = 0.744 and r^2^ = 0.917 and, for EGFR the values, were q^2^_LOO_ = 0.718 and r^2^ = 0.968.

**Figure 6:**
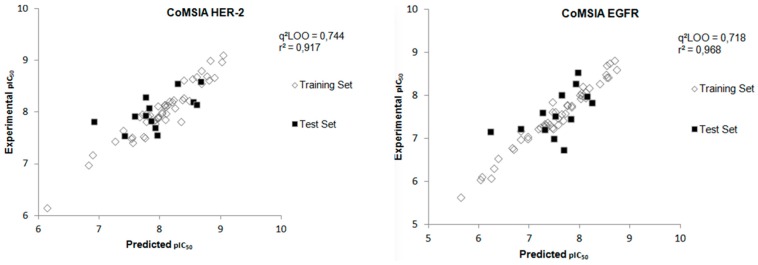
Experimental versus predicted pIC_50_ values for the training and test sets obtained from the CoMSIA model for both biological targets.

Using the best model generated for each target, the plots correlating experimental and predicted biological data were constructed, as shown in Figure 6.

After the construction of the best CoMSIA model using the compounds of the training set, the next step was to perform the external validation of this model using the test set, which contains 13 compounds that were not used in the construction phase of the model. Figure 6 shows the plot of the experimental and predicted pIC_50_ values by the CoMSIA model for the test set and Table 4 displays the values of experimental and predicted pIC_50_, as well as the residual values, for the test set obtained from the CoMSIA model for both biological targets. The external validation values show a good agreement between experimental and predicted pIC_50_ values.

**Figure 7:**
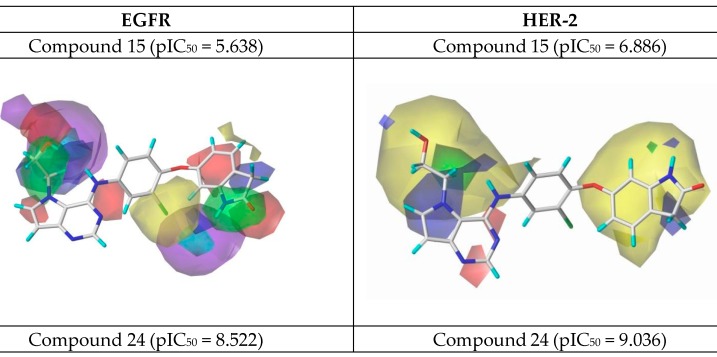

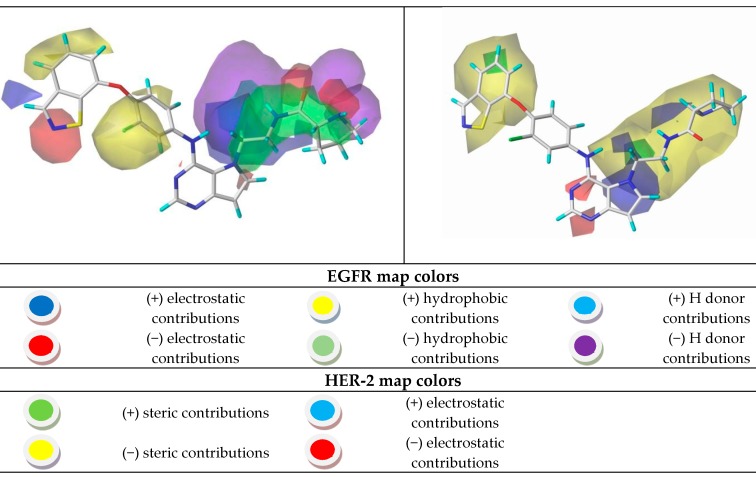
CoMSIA contour maps for the most and the least active compounds (EGFR and HER-2).

After the process of external validation, which confirmed the good predictive capacity of the best CoMSIA model obtained, 3D contour maps were generated. These maps allow the visualization of the regions with the main stereochemical, electrostatic, hydrophobic, hydrogen bond donor and hydrogen bond acceptor contributions. The 3D contour maps were generated for the most active ligand (24) and the least active one (15), as shown in Figure 7.

#### 2.2.3. New Compounds Proposed from CoMSIA Models

Using the results in Figure 7, we analyzed the electrostatic, hydrogen bonding, stereochemical and hydrophobic donor fields for the most and least active compounds (24 and 15, respectively). In HER-2 CoMSIA map, the blue fields suggest that substitutions by groups with positive charge density can be performed to improve the biological activity, and green fields suggest that bulky groups are well accepted. From the CoMSIA analyses for EGFR, blue fields also indicate substitutions by groups with positive charge density, yellow fields suggest substitutions related to hydrophobicity and cyan fields are related to contributions from hydrogen bonding donor atoms. Analyzing the most active compound (24), relative to HER-2, in the region of the ligand containing the ring with sulfur, the docking simulation was carried out precisely in the pocket of the active site possessing the non-polar residues, Met801 and Leu866, thus forming a site region of hydrophobic character. The pirrolidine group of the ligand is located at the entrance of the active site, which also consists of hydrophobic residues. However, according to the contribution maps, this region of the ligand presented steric problems for HER-2 and electrostatic ones for EGFR, in addition to the negative contributions to hydrogen bonding donor atoms.

**Figure 8:**
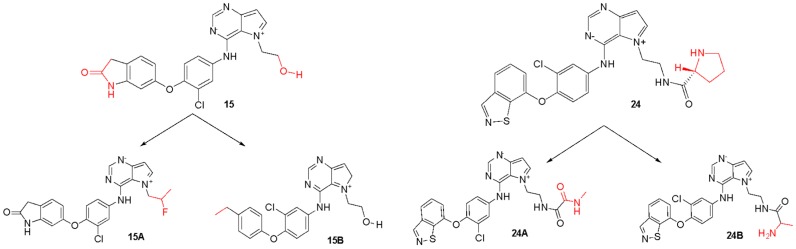
Original chemical structure of the compounds 15 and 24 and the new proposed compounds. (The red regions are groups that were substituted to compose the new molecules).

**Figure 9:**
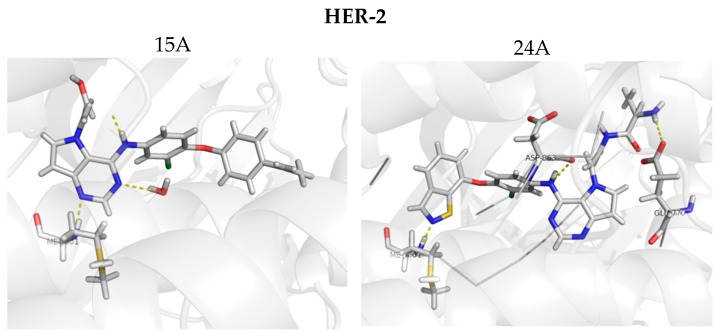

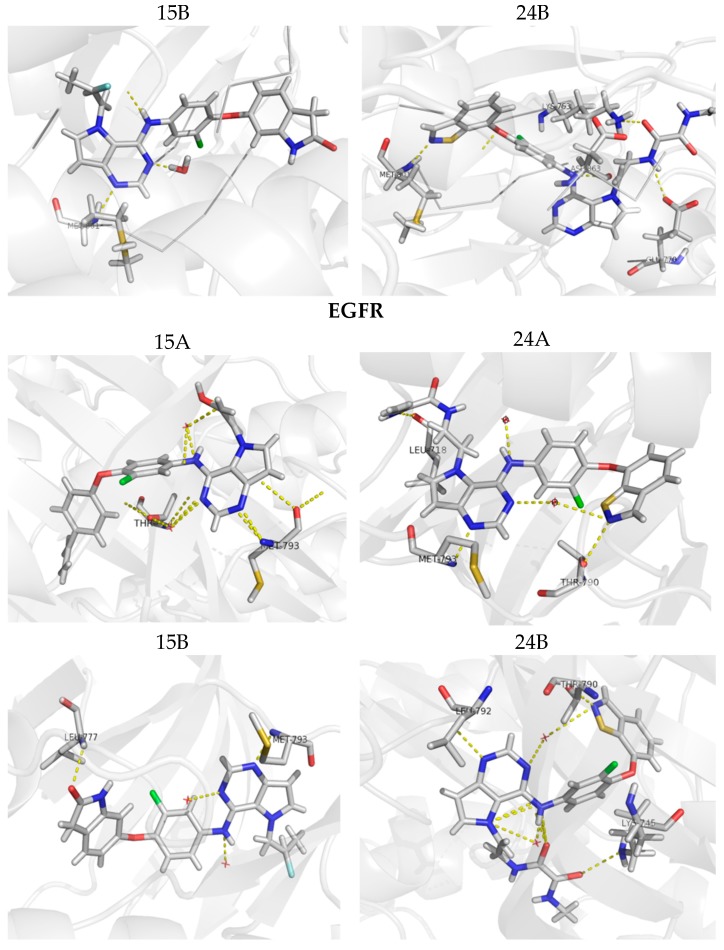
Molecular docking for the new proposed compounds (candidates to inhibit HER-2 and EGFR) from the CoMFA and CoMSIA models. The dash yellow lines indicate the interaction points between the proposed compounds and the main residues in the active sites.

For the least active compound, at the indolin-2-one region of the ligand, the maps pointed to poor hydrophobic regions close to oxygen and nitrogen, in addition to negative electrostatic contribution, for both targets. Thus, it is suggested a substitution of this region by more hydrophobic atoms/groups and with less steric hindrance to better fit the active site. In the hydroxyl region (n-ethan-1-ol), the maps suggest substitutions by less voluminous groups, pointing to problems of hydrophobicity and hydrogen bonding donors. From all the suggestions pointed by the CoMFA and CoMSIA models, we decided to propose new molecules as HER-2 and EGFR inhibitors and test them in our models. Figure 8 and Figure 9 illustrate the strategy used to propose the new compounds from the original chemical structure, as well as the molecular docking in the two studied targets using these compounds. In addition, four new compounds have been proposed, as shown in Table 5.

According to Table 5, it is possible to note that the original compound with the lowest biological activity (15) presented significant improvements from the substitutions suggested by the contribution maps, improving its pIC_50_ value from 5.638 to 7.518 for EGFR and 6.886 to 8.467 for HER-2.

Figure 9 shows the docking results obtained with the four new compounds proposed from the 3D models. Even the compounds that did not present improvements in the value of biological activity showed interactions with all of the main residues at the active site, suggesting a better fit and greater stability of the compounds in the site. The increasing in the number of interactions also confirms the improved biological activity of the proposed compounds (15A and 15B) when compared to the least active compound (15). We highlight compound 15B, which has an improvement in the original activity values from 5638 nM and 6886 nM to 7340 nM and 8467 nM for EGFR and HER-2, respectively. Moreover, the number of interactions is twice as large as the original compound, especially with Met793 and Met801, which are suggested as important to the stabilization of the inhibitors in the active site. From our analyses, it is possible to rationalize the fundamental interactions to inhibit EGFR and HER-2, as well as propose molecular modifications as a useful strategy to design new drug candidates with improved biological activity.

The pharmacokinetics and toxicity properties for the new proposed compounds were predicted using the online server pkCSM [22]. In addition, we compared the properties of the proposed molecules with two drugs available in the market: Lapatinib (a dual inhibitor of HER-2/EGFR) and Erlotinib (a EGFR inhibitor), calculated the same way. The obtained results (see Tables S1 and S2 in Supplementary Materials) showed that Lapatinib and Erlotinib are quite different according to the predicted toxicity data and the predicted toxicity data for the new designed compounds are similar to the mentioned drugs.

## 3. Discussion

Based on the receptor-based molecular alignment (Figure 6), we performed analyses of the results for the most (compound 24) and least active (compound 15) inhibitors of HER-2 and EGFR. The poses generated in the molecular docking can provide valuable information on the key ligand–receptor interactions related to the inhibition of EGFR and HER-2 receptors. Figure 3 shows that all compounds studied performed a polar interaction with Met793 at EGFR and Met801 at HER-2, which are important residues involved in the receptor–ligand crystallographic interactions. The most active compound at both targets has a large substituent group attached to the general structure, which provides a hydrophilic interaction between the aromatic system and both hydrophobic and hydrophilic pockets (according to Figure 2B). In addition, the most and the least active compounds (24 and 15, respectively) perform extra polar contact with Met793 (EGFR) and Met801 (HER-2). It is important to note that, for this active compound (24), the binding mode observed in the docking simulations is the same as that proposed by the authors who synthesized and tested the compounds [1,23,24]. Alternatively, the main interactions at the active site occur between this ligand and the following residues: Met793/Thr854 and Met801/Asp863 for EGFR and HER-2, respectively. Compound 15 (the least active) exhibits only the interaction with Thr854 and it does not interact with any of the major residues. In addition, compound 15 does not perform interactions in the hinge region of EGFR.

The most robust and predictive CoMFA model can also be used to rationalize the major ligand–receptor interactions from stereochemical and electrostatic contour maps. The stereochemical maps obtained from the CoMFA analyses are shown in Figure 8 and present favorable steric contributions in green and unfavorable steric contributions in yellow. Analyzing the electrostatic maps in Figure 8, we can see that, in the benzoisothiazole region, close to the nitrogen and sulfur atoms, substitutions by negative electrostatic groups are suggested, as well as *N*-hydroxypyrrolidine substituents. However, for compound 24 (the most active) at the HER-2 target, the region close to benzoisothiazole suggests a substitution by positive and negative groups. However, as in EGFR, the *N*-hydroxypyrrolidine region indicates favorable electrostatic interactions and, in fact, this region interacts with Asp863, which is a negatively charged polar residue. Compound 15 (the least active), considering its activity against EGFR, showed a favorable region in the ring containing nitrogen of the indolone group, whereas for HER-2, the electrostatic map suggests several modifications of negative character. In addition to the indolone region, close to the 7-(2-hydroxyethyl)-1-(phenylamine), the contour map also suggests substitutions by electronegative groups.

In relation to the stereochemical map, in the benzoisothiazole region, large green polyhedral are shown for both targets. These outlines indicate that, in these regions, substitutions by bulky groups can be carried out potentiating the biological activity of the compound. On the other hand, close to the 6-(2-chlorophenoxy) region, substitutions by less bulky groups are suggested. In the stereochemical analyses for both targets, it is possible to note that the maps suggest a substitution in the hydroxyl region by larger groups. Already, the position of the oxygen atom of 1-chloro-2-(cyclohexyloxy) indicates that less bulky groups would be more favorable.

## 4. Materials and Methods

### 4.1. Dataset

**Figure 10:**
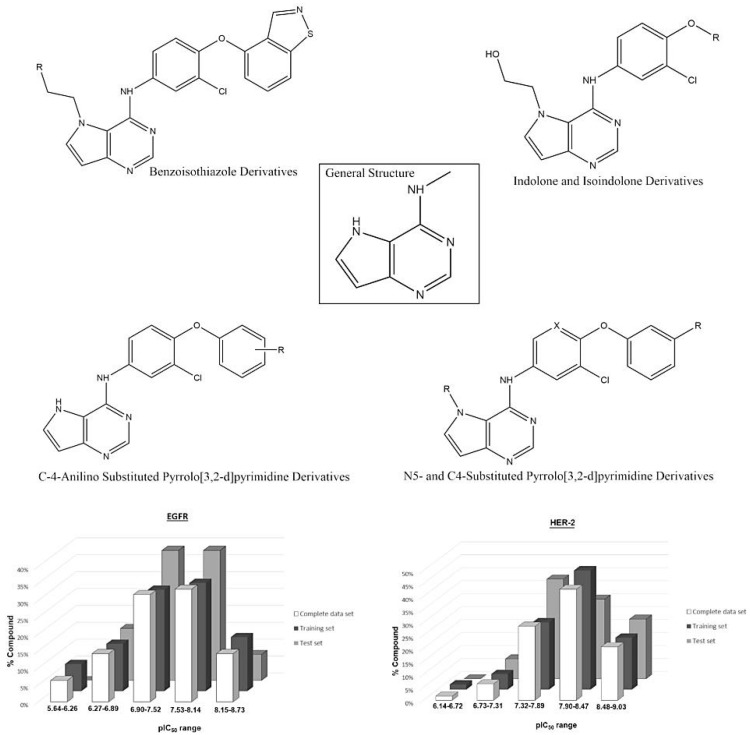
General structure of the studied compounds and some selected ligands, as well as the distribution of pIC_50_ for the training and test sets for each biological target.

A set containing 63 molecules with available biological data (IC_50_ values) was synthesized and tested in the same experimental conditions as Kawakita et al. and Ishikawa et al. [1,23,24]. These molecules comprised three different classes of diverse structures: pyrrolo[2,3-d]pyrimidine, heteroaromatic, indolone, isoindolone and benzoisothiazole derivatives, whose IC_50_ values were converted into pIC_50_ (−log IC_50_, see Tables S3 and S4 in Supplementary Materials). The chemical structures of the compound set and the range of the biological data guided the composition of the training and test sets. The external validation of the constructed models was made using the test set. Figure 10 shows some representative chemical structures in the compound set, including the most and least active molecules, and the distribution of pIC_50_ for all compound sets (training and test).

### 4.2. 3D Structure of EGFR and HER-2

**Figure 11:**
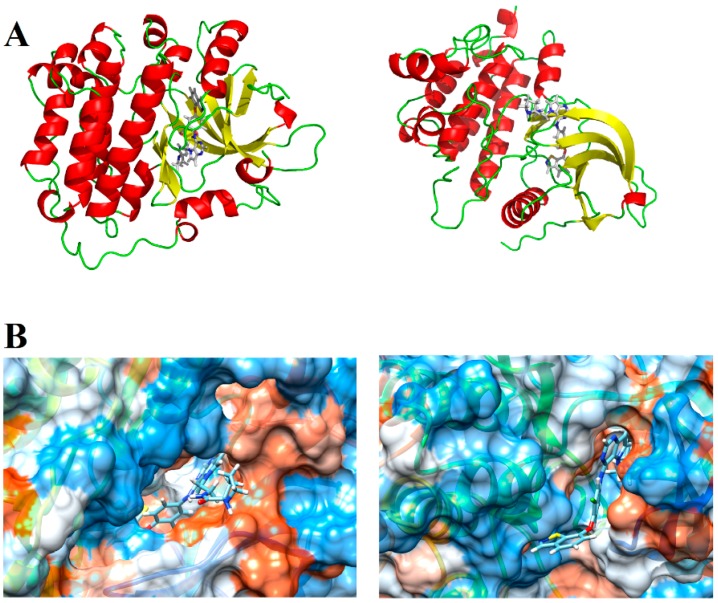
(**A**) Tridimensional structure of EGFR and HER-2; and (**B**) hydrophobic (orange) and hydrophilic (blue) surfaces of the EGFR and HER-2 active sites, respectively.

We then selected the structure of the biological targets studied (Figure 11A) to perform the docking analyses. The search for crystallographic structures of EGFR and HER-2 was performed at Protein Data Bank (PDB) (http://www.pdb.org/). We selected the structure of our targets based on some quality parameters (e.g., resolution, R-value and R-free values) and the chemical similarity between the crystallographic ligand and the compounds studied here. After various analyses, we decided to employ the EGFR structure with PDB ID = 3W32 (resolution = 1.80 Å, R-value and R-free equal to 0.236 and 0.200, respectively) and the HER-2 structure with PDB ID = 3PP0 (resolution = 2.25 Å, R-value and R-free equal to 0.260 and 0.185, respectively). We also generated a hydrophobic potential based on molecular surface of these receptors using USCF Chimera 1.5 (San Francisco, California, USA) [25]. Figure 11B shows the complete structure of EGFR and HER-2, as well the main polar interactions between the ligands and the binding site (generated by PyMOL software (The PyMOL Molecular Graphics System, Version 2.0 Schrödinger, LLC, New York, NY, USA), and the hydrophobic and hydrophilic surfaces of EGFR and HER-2 active site.

### 4.3. Redocking

Redocking analyses were performed since this approach aims to recover, from computational simulation, the original position of a ligand present in a crystallographic structure of a protein-ligand complex. Thus, redocking can be considered as a validation process [26] and, in this study, the crystallographic structure of the HER-2/EGFR already determined was used as reference. Thus, four different scoring functions were used to obtain the most similar ligand conformation to its experimental pose.

### 4.4. Conformational Sampling and Alignment

For the tridimensional quantitative study of the relationships between structure and activity related to the dual inhibitors of EGFR and HER-2, we employed the technique known as comparative molecular field analysis (CoMFA) [27] and comparative molecular similarity index analysis (CoMSIA). The three-dimensional alignment of the compound set is a prerequisite for CoMFA and CoMSIA techniques and molecular docking is one of several ways to make this, as used in some studies (e.g., [28,29]). To perform the docking simulations, the selected PDB structures were prepared by adding hydrogens and charges, retaining some important water molecules (“2001; 2010” and “22”, for EGFR and HER-2, respectively), and excluding all others and all ligands. A radius sphere of 5 Å was defined around the crystallographic ligand as the docking target site.

Molecular docking was performed taking into account the rigid protein structure and the flexibility of some important residues at the active site, as well as the flexibility of the ligands, using GOLD program (Cambridge, UK) [30]. Each ligand pose was obtained considering Goldscore (scoring function) values for HER-2 and Chemscore for EGFR, and the main interactions described in the literature. Therefore, at the end of the docking simulations, the molecular alignment was obtained for each biological target, which is required for the analyses of the tridimensional quantitative relationships between structure and activity of the dual inhibitor series.

### 4.5. CoMFA and CoMSIA Analyzes

After the docking simulations, we calculated the atomic charges for all aligned compounds, employing the semi-empirical method PM3 [31,32] at the MOPAC program (Colorado Springs, CO, USA). Then, we generated the three-dimensional CoMFA models [33] based on electrostatic and stereochemical molecular interaction fields (MIFs). To optimize the CoMFA models, the option “region focusing” was employed which is based on the distance of 3D grid points (0.5–2.0) and standard deviation of calculated descriptors (0.3–0.7). Partial Least Squares (PLS) regression was employed to generate statistically significant models. All these programs are implemented in the package Sybyl 8.1 (Saint Louis, Missouri, USA).

In addition to the CoMFA analyses, this study also used the CoMSIA technique. In this approach, besides the electrostatic and stereochemical fields, it is possible to analyze the hydrophobic fields, hydrogen bond donors and acceptors. In this way, the CoMSIA model was generated using the same alignment obtained with the total set used in the CoMFA method. Next, the CoMSIA fields were combined in all possible ways, and the focusing technique was employed following the same parameters used with the CoMFA technique to generate the best model. Furthermore, statistical validations were performed to corroborate the quality of the models, as well as 3D contour maps were generated to indicate possible molecular modifications with the aim of proposing new candidates to inhibit EGFR and HER-2.

## 5. Conclusions

Protein kinases (in this case, HER-2 and EGFR) are involved in cancer-related diseases. Due to the deregulation of genes that control cell growth, substances that inhibit protein kinases can be employed in the treatment of breast cancer, for example. In this study, a set containing 63 dual inhibitors of HER-2 and EGFR was analyzed with the following computational approaches: molecular docking (to obtain the structural alignment at each biological target), CoMFA and CoMSIA analyses. The models obtained using these techniques presented good predictive capacity, since the internal and external validations carried out showed that these models have a good correlation between the computationally predicted and the experimental biological values.

From the CoMFA analyses, it was possible to verify that the model showed a good statistical quality for HER-2 (q^2^_LOO_ = 0.827) and EGFR (q^2^_LOO_ = 0.728). To evaluate the predictive power of the best models generated, external validation was performed using the test compounds. The significant agreement between the predicted and predicted pIC_50_ values for HER-2 (r^2^_pred_ = 0.999) and EGFR (r^2^_pred_ = 0.998) indicate the predictive capacity of the CoMFA models. For the CoMSIA models, it was verified that the model has statistical quality for HER-2 (q^2^_LOO_ = 0.744) and EGFR (q^2^_LOO_ = 0.718), indicating the predictive capacity of the CoMSIA models. After the statistical analyses and its validations, the CoMFA and CoMSIA contour maps of the most and the least active compounds at each target were analyzed, indicating the regions with better contributions to the biological activity. The analysis of these maps suggested substitutions by groups that can potentiate the biological activity of new compounds, where four new compounds were proposed, prioritizing the contribution map of each biological target, for the most and least active compounds. From this, the new compounds were aligned to the CoMFA and CoMSIA models and the values of biological activity were predicted, showing significant improvements, mainly for the least active compound. Therefore, the results obtained in this study indicate the main interactions that occur between the inhibitors studied and the biological targets and may help the proposition of new potential dual inhibitors of HER-2 and EGFR, candidates to treat breast cancer.

## Figures and Tables

**Table 1:** Results obtained for the CoMFA standard model and the best focused models for HER-2 and EGFR.

Statistical Parameter	HER-2		EGFR	
	No Focus	*w* = 0.5/*d* = 1.0	No Focus	*w* = 0.5/*d* = 1.0
q^2^_LOO_	0.492	0.827	0.043	0.728
r^2^	0.986	0.982	0.966	0.973
SEE	0.102	0.078	0.141	0.126
SEP	0.394	0.235	0.804	0.391
E	0.593	0.585	0.400	0.367
S	0.407	0.415	0.600	0.633
N	3	5	5	4

q^2^_LOO_, Coefficient of validation using leave-one-out method; SEP, standard error of prediction; N, number of principal components generated from PLS; r^2^, coefficient of correlation without validation; SEE, standard error of estimation; S, contribution of steric fields; E, contribution of electrostatic fields; *w*, standard deviation weight; *d*, grid distance factor (Å).

**Table 2:** Values of experimental and predicted pIC_50_, and residual values for the test set obtained from the CoMFA model for both biological targets.

Compound	HER-2			EGFR		
	Experimental pIC_50_	Predicted pIC_50_	Residual	Experimental pIC_50_	Predicted pIC_50_	Residual
51	7.824	7.827	−0.003	7.481	7.456	0.024
52	7.921	7.904	0.016	7.420	7.437	−0.017
53	7.959	7.967	−0.008	7.959	7.946	0.012
54	6.921	6.927	−0.006	6.823	6.813	0.010
55	7.585	7.579	0.005	6.537	6.547	−0.009
56	8.678	8.676	0.001	8.244	8.244	−0.007
57	8.292	8.281	0.011	7.823	7.830	−0.006
58	8.553	8.567	−0.014	8.142	8.148	−0.006
59	7.770	7.771	−0.001	7.301	7.295	0.005
60	7.854	7.854	0.000	7.301	7.256	−0.004
61	7.021	7.017	0.003	7.508	7.505	0.003
62	6.824	6.826	−0.002	6.050	6.053	−0.002
63	8.602	8.602	−0.000	7.677	7.679	−0.001

**Table 3:** Results for the best CoMSIA model (HER-2 and EGFR) using no focus and focus techniques.

Statistical Parameter	HER-2		Parameter Statistical	EGFR	
	No Focus	*w* = 0.5/*d* = 1.0		No Focus	*w* = 0.3/*d* = 1.0
q^2^_LOO_	0.502	0.744	q^2^_LOO_	0.457	0.718
r^2^	0.942	0.917	r^2^	0.975	0.968
SEE	0.144	0.173	SEE	0.125	0.144
SEP	0.410	0.304	SEP	0.589	0.433
E	0.716	0.651	E	0.415	0.459
S	0.284	0.349	H	0.187	0.245
D	-	-	D	0.398	0.296
N	3	6	N	6	6

q^2^_LOO_, Validation coefficient using the “one-out” method; SEP, standard error of prediction; N, number of main coefficients generated from PLS; r^2^, regression coefficient without cross validation; SEE, standard non-cross validation error; S, stereochemical contributions; E, electrostatic contributions; H, hydrophobic contributions; D, contribution of hydrogen bonding donors; A, contribution of hydrogen bond acceptors.

**Table 4:** Values of experimental and predicted pIC_50_, and the residual values, for the test set obtained from the CoMSIA model for both biological targets.

Compound	HER-2			EGFR		
	Experimental pIC_50_	Predicted pIC_50_	Residual	Experimental pIC_50_	Predicted pIC_50_	Residual
51	7.823	8.083	−0.260	7.481	6.990	0.491
52	7.921	7.693	0.228	7.509	7.524	−0.015
53	7.959	7.066	0.893	7.959	8.526	−0.567
54	6.921	7.810	−0.889	6.824	7.222	−0.398
55	7.585	7.921	−0.336	6.229	7.164	−0.935
56	8.678	8.584	0.094	8.244	7.837	0.407
57	8.292	8.545	−0.253	7.824	7.455	0.369
58	8.553	8.195	0.358	8.142	7.984	0.158
59	7.770	7.936	−0.166	7.638	8.010	−0.372
60	7.854	7.829	0.025	7.252	7.601	−0.349
61	7.420	7.542	−0.122	7.921	8.270	−0.349
62	7.770	8.295	−0.525	7.301	7.200	0.101
63	8.602	8.141	0.461	7.678	6.733	0.945

**Table 5:** New compounds proposed from the 3D models and the predicted pIC_50_ values.

Original Compound	Experimental pIC_50_		Modified Compound	pIC_50_ Predicted	
	EGFR Activity	HER-2 Activity		EGFR Activity	HER-2 Activity
15	5.638	6.886	15A	7.518	6.356
			15B	7.340	8.467
24	8.523	9.036	24A	7.744	8.126
			24B	7.658	8.027

## Supplementary Materials

Supplementary Materials can be found at http://www.mdpi.com/1422-0067/19/12/3728/s1.

## Abbreviations

CoMFA	Comparative molecular field analysis
CoMSIA	Comparative molecular similarity index analysis
EGFR	Epidermal growth factor receptor
HER-2	Human Epidermal growth factor Receptor 2
PLS	Partial least squares regression
q^2^	Cross-validated correlation coefficient
q^2^_LOO_	Coefficient of validation using leave-one-out method
r^2^	Non-cross-validated correlation coefficient
RMSD	Root mean square deviation
SEE	standard error of estimation
SEP	Standard error of prediction
