# Supplementary material for: Studies on the Dual Activity of EGFR and HER-2 Inhibitors Using Structure-Based Drug Design Techniques
Source: Int J Mol Sci. 2018 Nov 23;19(12):3728. doi: 10.3390/ijms19123728 (PMC6321113; doi:10.3390/ijms19123728)
Supplement: Supplementary file 1 [file ijms-19-03728-s001.pdf]

Supplementary Material

Table S1 – Prediction of absorption (A), distribution (D), metabolism (M) and excretion (E) for the new proposed compounds and some commercial available drugs.

Property	Model Name	Predicted Value					
		Lapatinib	Erlotinib	15A	15B	24A	24B
A	Water solubility (log mol/L)	-4.324	-4.403	-4.75	-4.594	-4.006	-3.746
A	Caco2 permeability (log Papp in 10^{-6} cm/s)	-0.402	1.238	0.975	0.862	0.699	0.761
A	Intestinal absorption (human) (% Absorbed)	100	95.549	93.918	93.552	81.608	80.580
A	Skin Permeability (log Kp)	-2.735	-2.738	-3.299	-2.747	-2.904	-2.884
A	P-glycoprotein substrate	Yes	No	Yes	Yes	Yes	Yes
A	P-glycoprotein I inhibitor	Yes	Yes	Yes	Yes	Yes	Yes
A	P-glycoprotein II inhibitor	Yes	Yes	No	Yes	No	No
D	VDss (human) (log L/kg)	-0.221	-0.053	0.251	0.381	0.010	0.682
D	Fraction unbound (human) (FU)	0.153	0.04	0.133	0.148	0.124	0.226
D	BBB permeability (log BB)	-0.785	-0.67	-0.682	-0.796	-1.126	-1.217
D	CNS permeability (log PS)	-3.124	-3.384	-3.062	-2.198	-3.286	-3.624
M	CYP2D6 substrate	No	No	No	No	No	No
M	CYP3A4 substrate	Yes	Yes	Yes	Yes	Yes	Yes
M	CYP1A2 inhibitor	No	Yes	No	Yes	No	No
M	CYP2C19 inhibitor	Yes	Yes	Yes	Yes	No	No
M	CYP2C9 inhibitor	Yes	Yes	Yes	Yes	No	No
M	CYP2D6 inhibitor	No	No	No	No	No	No
M	CYP3A4 inhibitor	Yes	Yes	Yes	Yes	No	Yes
E	Total Clearance (log ml/min/kg)	0.542	0.591	0.052	0.164	-0.023	0.463
E	Renal OCT2 substrate	Yes	No	No	No	No	No

Table S2 – Prediction of toxicity for the new proposed compounds and some commercial available drugs.

Model Name	Predicted Value					
	**Lapatinib**	**Erlotinib**	**15A**	**15B**	**24A**	**24B**
AMES toxicity	Yes	No	No	No	No	No
Max. tolerated dose (human) (log mg/kg/day)	0.436	0.002	-0.843	0.308	-0.300	-0.331
hERG I inhibitor	No	No	No	No	No	No
hERG II inhibitor	Yes	Yes	Yes	Yes	Yes	Yes
Oral Rat Acute Toxicity (LD50) (mol/kg)	3.171	2.368	3.272	2.959	2.423	2.694
Oral Rat Chronic Toxicity (LOAEL) (log mg/kg_bw/day)	0.064	0.880	0.021	0.836	1.659	1.286
Hepatotoxicity	Yes	Yes	Yes	Yes	Yes	Yes
Skin Sensitisation	No	No	No	No	No	No
T.Pyriformis toxicity (log ug/L)	0.285	0.334	0.383	0.319	0.291	0.298
Minnow toxicity (log mM)	-2.281	-0.437	0.640	0.134	0.969	2.339

Table S3 - Structure and biological data of the compounds in the training set.

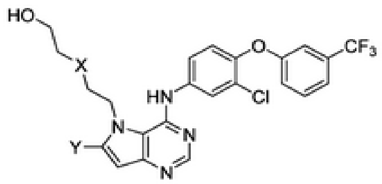

Compound	X	Y	pIC_{50} - HER2	pIC_{50} - EGFR
**1**	NH	H	8.114	7.657
**2**	S	H	8.027	7.356
**3**	SO_2	H	7.921	7.397
**4**	O	CN	7.721	6.638

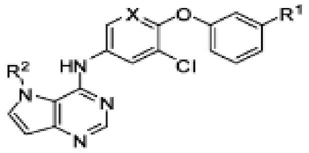

Compound	X	R^1	R^2	pIC_{50} - HER2	pIC_{50} - EGFR
**5**	CH	Cl	CH_3	8.481	8.036
**6**	CH	CF_3		7.959	7.568
**7**	CH	Cl	2-hydroxy ethyl	8.387	8.022
**8**	CH	CF_3		7.921	7.468
**9**	CH	OCF_3		7.538	6.958
**10**	CH	OCF_3		8.076	7.309
**11**	N	CF_3		7.959	6.376

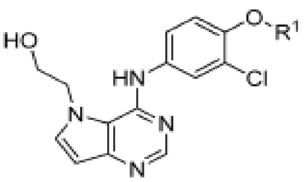

Compound	R¹	pIC₅₀ - HER2	pIC₅₀ - EGFR
**12**	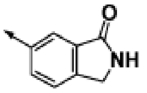	8.886	8.108
**13**	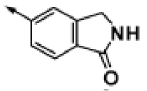	7.745	6.292
**14**	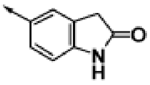	7.553	6.017
**15**	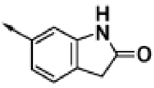	6.886	5.638
**16**	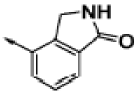	6.824	6.050
**17**	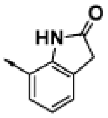	8.824	8.552
**18**	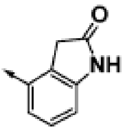	8.796	8.522
**19**	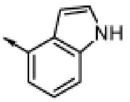	8,602	8,508
**20**	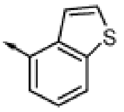	8.222	8.055
**21**	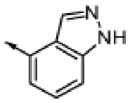	8.770	8.721
**22**	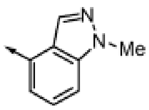	8.678	7.443
**23**	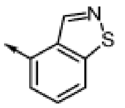	8.678	8.387

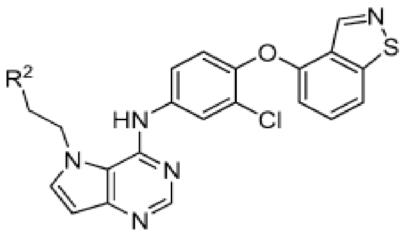

Compound	R^1	pIC_{50} - HER2	pIC_{50} - EGFR
**24**	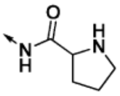	9.036	8.522
**25**	-NHCOC(CH_3)$_2$$NH_2$	9.009	8.585

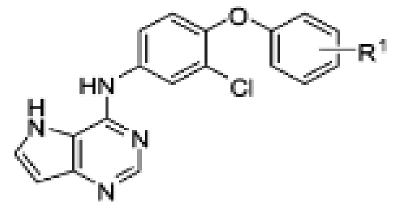

Compound	R^1	pIC_{50} - HER2	pIC_{50} - EGFR
**26**	H	7.796	6.958
**27**	2-F	8.032	7.214
**28**	3-F	7.699	6.823
**29**	2-Cl	7.959	7.568
**30**	3-Cl	8.081	7.455
**31**	2-CN	8.097	7.292
**32**	3-CN	8.086	6.823
**33**	4-CN	6.143	6.229
**34**	3-CF_3	7.523	7.173
**35**	2-OCF_3	7.260	6.677
**36**	3-OCF_3	7.387	6.657
**37**	3-CH_3	8.337	7.698
**38**	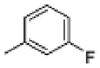	7.886	8.113
**39**	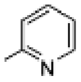	7.658	7.744

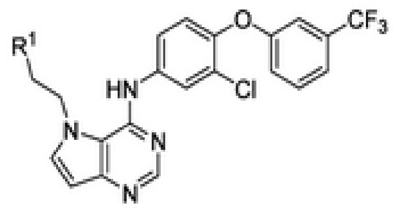

Compound	R^1	pIC_{50} - HER2	pIC_{50} - EGFR
**40**	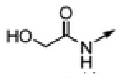	8.367	8.000
**41**	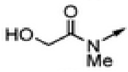	8.244	7.823
**42**	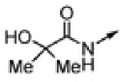	7.824	7.455

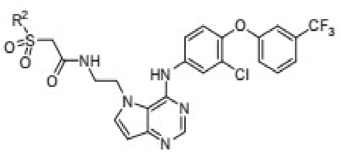

Compound	R^2	pIC_{50} - HER2	pIC_{50} - EGFR
**43**	Me	8.201	7.823
**44**	Et	8.071	7.508
**45**	Pr	7.959	7.275
**46**		7.770	7.638

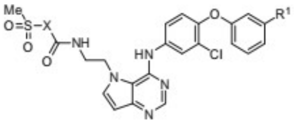

Compound	X	R^1	pIC_{50} - HER2	pIC_{50} - EGFR
**47**	CH(Me)	CF_3	8.149	7.744
**48**	$C(Me)_2$	CF_3	7.770	7.327
**49**	CH_2	Cl	8.538	8.173
**50**	CH(Me)	Cl	8.387	8.008

Table S4 - Structure and biological data of the compounds in the test set.

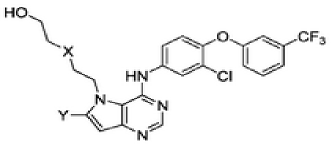

Compound	X	Y	pIC_{50} - HER2	pIC_{50} - EGFR
**51**	CH_2	H	15	33
**52**	SO	H	12	31
**53**	O	CH_3	12	38

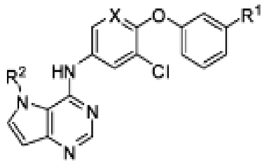

Compound	X	R1	R2	pIC_{50} - HER2	pIC_{50} - EGFR
**54**	CH	OCF_3		17	50
**55**	CH		2-(2-hydroxy ethoxy) ethyl	2,1	5,7
**56**	CH	CF3		5,1	15

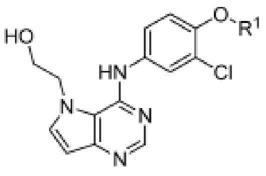

Compound	R^1	pIC_{50} - HER2	pIC_{50} - EGFR
**57**	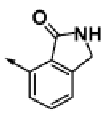	2,5	21

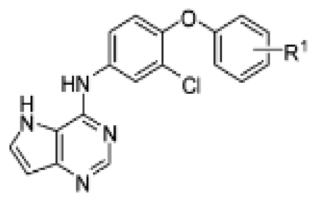

Compound	R^1	pIC_{50} - HER2	pIC_{50} - EGFR
**58**	2-CF3	120	150
**59**	3-OCH3	26	290

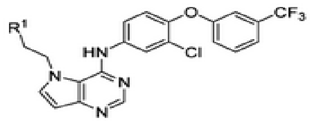

Compound	R^1	pIC_{50} - HER2	pIC_{50} - EGFR
**60**	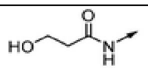	8.553	8.142
**61**	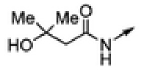	7.770	7.638

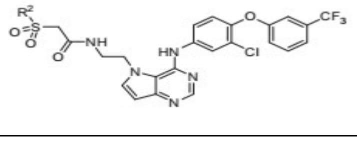

Compound	R^2	pIC_{50} - HER2	pIC_{50} - EGFR
**62**	Bu	7.854	7.251

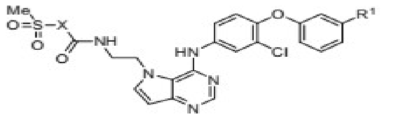

Compound	X	R^1	pIC_{50} - HER2	pIC_{50} - EGFR
**63**	$C(Me)_2$	Cl	7.959	7.959

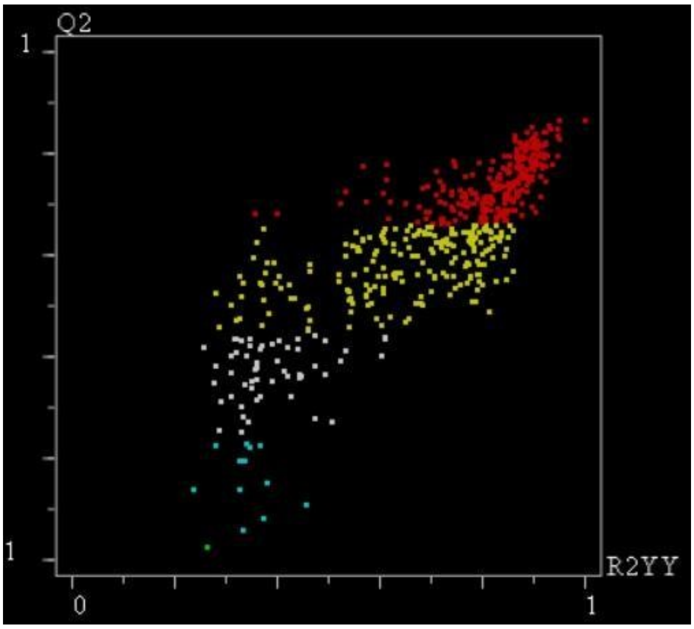

(A)

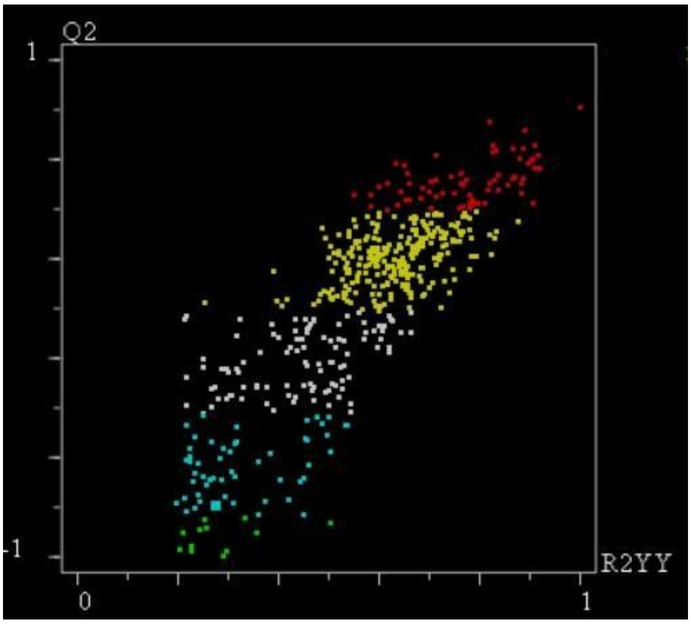

(B)

Figure S1. Results obtained from the scrambling tests for (A) HER-2 and (B) EGFR.
